# *Pseudomonas stutzeri* and *Kushneria marisflavi* Alleviate Salinity Stress-Associated Damages in Barley, Lettuce, and Sunflower

**DOI:** 10.3389/fmicb.2022.788893

**Published:** 2022-03-08

**Authors:** Sonia Szymańska, Marta Izabela Lis, Agnieszka Piernik, Katarzyna Hrynkiewicz

**Affiliations:** ^1^Department of Microbiology, Faculty of Biological and Veterinary Sciences, Nicolaus Copernicus University, Toruń, Poland; ^2^Department of Geobotany and Landscape Planning, Faculty of Biological and Veterinary Sciences, Nicolaus Copernicus University, Toruń, Poland

**Keywords:** salinity, growth parameters, crop plants, *Hordeum vulgare*, *Lactuca sativa*, *Heliantus annuus*, endophytes

## Abstract

Soil salinity is one of the most important abiotic factors limiting plant productivity. The aim of this study was to determine the effect of selected halotolerant plant growth-promoting endophytes (PGPEs, *Pseudomonas stutzeri* ISE12 and *Kushneria marisflavi* CSE9) on the growth parameters of barley (*Hordeum vulgare*), lettuce (*Lactuca sativa*), and sunflower (*Helianthus annuus*) cultivated under salt stress conditions. A negative effect of two higher tested salinities (150 and 300 mM NaCl) was observed on the growth parameters of all investigated plants, including germination percentage and index (decreasing compared to the non-saline control variant in the ranges 5.3–91.7 and 13.6–90.9%, respectively), number of leaves (2.2–39.2%), fresh weight (24.2–81.6%); however, differences in salt stress tolerance among the investigated crops were observed (*H. annuus* > *H. vulgare* > *L. sativa*). Our data showed that the most crucial traits affected by endophyte inoculation under salt stress were chlorophyll concentration, leaf development, water storage, root development, and biomass accumulation. Thus, the influence of endophytes was species specific. *K. marisflavi* CSE9 promoted the growth of all tested plant species and could be considered a universal PGPEs for many plant genotypes cultivated under saline conditions (e.g., increasing of fresh weight compared to the non-inoculated control variant of barley, lettuce, and sunflower in the ranges 11.4–246.8, 118.9–201.2, and 16.4–77.7%, respectively). *P. stutzeri* ISE12 stimulated growth and mitigated salinity stress only in the case of barley. Bioaugmentation of crops with halotolerant bacterial strains can alleviate salt stress and promote plant growth; however, the selection of compatible strains and the verification of universal plant stress indicators are the key factors.

## Introduction

The increasing world population and associated rising demand for agricultural products continually contribute to soil degradation (Adejumobi et al., 2016; Temme et al., 2019) and soil salinization is one of the most critical consequences. Saline areas occur in at least 100 countries and cover 932.2 Mha worldwide (Cuevas et al., 2019). Soil salinization may result from human activities, such as improper irrigation, deforestation and intensive cropping, and natural factors, e.g., mineral weathering and soil derived from salt-affected rocks (Athar and Ashraf, 2009; Srinivasarao et al., 2021). Due to the level of salinity, soils have been divided into non-saline (ECe 0–2 dS/m), slightly saline (ECe 2–4 dS/m), moderately saline (ECe 4–8 dS/m), strongly saline (ECe 8–16 dS/m), and extremely saline (ECe > 16 dS/m) (Corwin and Scudiero, 2019). Increasing soil salinization decreases seed germination, plant growth and development, water and nutrient uptake, and physiological (e.g., photosynthesis) and biochemical processes of plants (Isayenkov and Maathuis, 2019; Kumar et al., 2020). The tolerance level of plants to salinity may depend on the species, cultivar and growth phase (Munns and Tester, 2008; Rajabi Dehnavi et al., 2020). Only a small group of plants, known as halophytes, have the ability to grow in salt conditions exceeding 200 mM NaCl, while the vast majority of plants are in the group of glycophytes (including most crops) that cannot accumulate salts in plant tissues but are able to survive in saline environments (Carillo et al., 2011; Batool et al., 2014; Guo et al., 2021).

Plant species belonging to the glycophytes can exhibit different levels of tolerance to salt stress (Green et al., 2017). As representatives of the *Asteraceae* family, lettuce (*Lactuca sativa* L.) and sunflower (*Helianthus annuus* L.) are known as moderately salt-sensitive plants (tolerating 50–150 mM NaCl) (Isayenkov and Maathuis, 2019; Temme et al., 2019). Lettuce is a commonly grown vegetable because of its taste and high contents of antioxidants, vitamins (A, C, B9, and K), minerals (calcium, phosphorus, potassium, manganese, and iron) and fiber (Sapkota et al., 2019; Azarmi-Atajan and Sayyari-Zohan, 2020). Sunflower is the third most important source of edible vegetable oil worldwide and an efficient source of biodiesel; it also has other applications, e.g., as medicines, nourishments, feedstocks, fodders, decorations, and phytoremediators (Talia et al., 2011; Fernández-Luqueño et al., 2014; Ittah et al., 2019). Barley (*Hordeum vulgare* L.), representative of the *Poaceae* family, is one of the most salt stress tolerant crops among glycophytes and is considered the most adaptable cereal to salinity (El Madidi et al., 2006; Zhou, 2009; Adjel et al., 2013). Barley is a popular crop and is planted in a wide range of environments worldwide, including the Mediterranean, oceanic and continental climates, as well as in arctic and subarctic zones. The economic importance of this crop is similar to that of wheat and rice, taking fourth place in quantity and area of cultivation (Zhou, 2009; Dawson et al., 2015). This cereal is a great source of food and drink for humans, and it is also used for feeding livestock (FAOSTAT, 2014; Mohammed et al., 2021).

The application of halotolerant plant growth-promoting endophytes (PGPEs) isolated from halophytes, e.g., *Salicornia europaea*, seems to be an appropriate solution in sustainable agriculture (Davy et al., 2001; Furtado et al., 2019a,b; Szymańska et al., 2019; Christakis et al., 2021; El-Tarabily et al., 2021). Endophytes (microorganisms living within plant tissues without causing any negative effect on host plants) associated with crops can directly (e.g., nitrogen fixation and phytohormone and siderophore synthesis) and/or indirectly (e.g., protection against pests and herbivores) stimulate plant growth and protect them against unfavorable environmental conditions (Khan et al., 2016; Rabiey et al., 2019; Eid et al., 2021). In our previous publications, we confirmed the positive effect of the halotolerant bacterium *P. stutzeri* ISE12 isolated from *S. europaea* on beetroot (*Beta vulgaris*) (Piernik et al., 2017; Szymańska et al., 2020) and canola (*Brassica napus* L.) (Szymańska et al., 2019) from the Amaranthaceae and Brassicaceae families, respectively.

In the present work, we hypothesized that the halotolerant endophytic bacterial strains *P. stutzeri* ISE12 and *K. marisflavi* CSE9 isolated from the obligatory halophyte *S. europaea* will alleviate the salt stress of three tested plant species (*H. vulgare*, *L. sativa*, and *H. annuus*). The main objective of the current work was to evaluate: (i) the tolerance of three crops (*H. vulgare*, *L. sativa*, and *H. annuus*) to increasing salinity, (ii) the effect of inoculation with *P. stutzeri* ISE12 or *K. marisflavi* CSE9 on the plant growth parameters of *H. vulgare*, *L. sativa*, and *H. annuus* growing under different salt stress conditions (0, 50, 150, 300 mM NaCl) and (iii) the selection criteria of universal plant growth parameters susceptible to changes caused by salinity and inoculation.

## Materials and Methods

### Experimental Design

**FIGURE 1:**
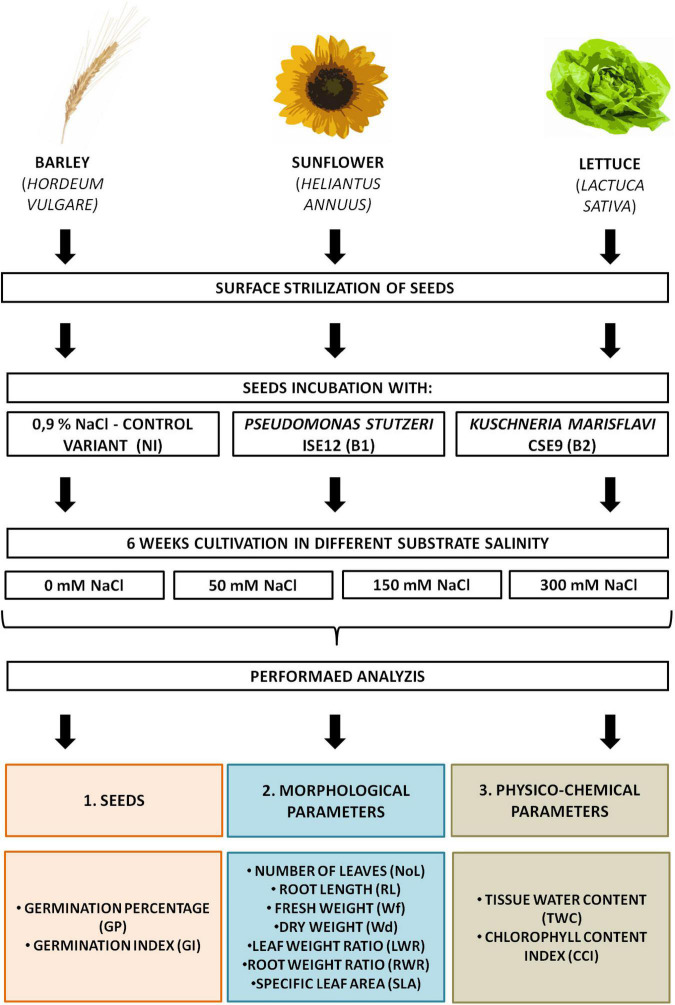
Experimental design.

In the experiment, we investigated three crops with different salt tolerance properties: sunflower (*H. annuus*), lettuce (*L. sativa*), and barley (*H. vulgare*). Commercial seeds of sunflower (*H. annuus*) and butterhead lettuce (*L. sativa*) were purchased from Torseed (Toruń, Poland) and Plantico Company (Stare Babice, Poland), while winter barley (*H. vulgare*) seeds were obtained from the Plant Breeding and Acclimatization Institute (IHAR) (National Research Institute, Strzelce, Poland). Healthy and uniformly sized seeds were surface sterilized using a 30% hydrogen peroxide and 70% ethanol mixture (1:1, final concentration 15% hydrogen peroxide and 35% ethanol) for 11 (sunflower), 7 (lettuce), and 3.5 min (barley). Then, seeds were rinsed six times with sterile distilled water. The residual water after the last washing was used for the sterility test for bacteria and fungi, by plating on R2A (Difco™ and BBL™) and PDA (Difco™ and BBL™) plates, respectively. Only successfully sterilized seeds were used in the pot experiment. For each investigated plant species, three inoculation variations were used: control (non-inoculated, NI) and inoculated with one of two endophytic bacterial (EB) strains (B1—*P. stutzeri* ISE12 and B2—*K. marisflavi* CSE9). Plants were cultivated in one of 4 salinity treatments: 0, 50, 150, or 300 mM. Substrate (sterile mixture of sand and vermiculite, 1: 1) in plastic tray (each pot: 30 × 46 cm, 0.077 L) was salted by adding to the litter box 2 l of sterile Hoagland’s medium enriched with the appropriate concentration of NaCl (Figure 1). Seeds were sown into growth substrate and cultivated under controlled conditions (temperature: 24 ± 2°C and light/dark: 16/8 h) and were irrigated with Hoagland medium (once per week) and distilled water (according to plant requirements) (Hoagland and Arnon, 1950). After 6 weeks, plants were collected and analyzed (Figure 1).

### Characterization of Bacterial Strains and Inoculation Procedure

Two halotolerant endophytic bacterial strains, *P. stutzeri* ISE12 (B1) and *K. marisflavi* CSE9 (B2), were used for the inoculation of plants in the pot experiment. Both bacterial strains were originally isolated from the roots of *S. europaea* and characterized for plant growth promoting (PGP) properties (Szymańska et al., 2016, 2020). *P. stutzeri* ISE12 (NCBI Acc. No. KX686983) was characterized as having a wider range of plant growth-promoting potential (e.g., nitrogen fixation, siderophores, IAA synthesis) and lower tolerance to salinity than *K. marisflavi* CSE9 (NCBI Acc. No. KX027360) (Szymańska et al., 2016, 2020).

Bacterial inocula were prepared using cells suspended in 2% NaCl solution, which was then diluted to OD = 0.5 (OD—optical density, measured at 600 nm, equivalent to 1.5 × 10^8^ cells/ml) after 72 h of culture incubation on R2A medium (Difco™) supplemented with 2% NaCl at 24°C. Plants cultivated in the pot experiment were inoculated with bacterial strains twice: 1st inoculation—surface sterilized seeds were incubated in bacterial inocula for 45 min at 24°C with shaking (control variant—non-inoculated seeds were incubated in sterile 0.9% NaCl solution); 2nd inoculation—1 ml of bacterial inocula was added to the growth substrate close to the root zone of 2-week-old seedlings (control variant—2% NaCl solution was used).

### Growth Assessments

The number of germinated seeds was determined daily (at 10 a.m.) for 10 days.

The germination percentage (GP) was calculated according to the International Seed Testing Association (ISTA) method:

GP = number of normally germinated seeds/total number of seeds sown × 100

The germination index (GI) was calculated using the following formula:


(1) GI=Σ⁢(Gt/Tt),

where Gt is the number of seeds germinated on day t, and Tt is the number of days (Ruan et al., 2002).

Chlorophyll levels in leaves were detected by a hand-held Chl meter CCM-200 (Opti-Sciences, Tyngsboro, Massachusetts, United States) to assess the chlorophyll content index (CCI) after 6 weeks of plant cultivation (Richardson et al., 2002). Then, the plants were carefully removed from the pots, and the roots were washed using tap water to eliminate the residue from the growth substrate. The number of leaves and root and shoot lengths of 42-day-old plants were measured. Leaf area was scanned and measured by digiShape software.

The tissue water content (TWC) was calculated according to the formula of Black and Pritchard (2002):


(2) TWC=(W-fW)d/W100f*.

Biomass accumulation was calculated as fresh (W_f_) and dry weight (W_d_). The dry weight (W_d_) was assessed after 72 h of drying at 85°C. Moreover, the following growth indices were calculated: specific leaf area (SLA), leaf weight ratio (LWR), and root weight ratio (RWR) (Piernik et al., 2017).

### Statistical Analysis

Two-way ANOVA was used to check the effect of EB inoculation and NaCl concentration on growth parameters of tested plants. One-way ANOVA was used with Tukey’s *post hoc* test to compare differences between treatments. Salt resistance assessments were based on comparisons with NI conditions by one-way ANOVA with Tukey’s *post hoc* test. General assessment of the effect of inoculation (*P. stutzeri* ISE12—B1 and *K. marisflavi* CSE9—B2) on the growth of *H. vulgare*, *L. sativa*, and *H. annuus* was performed using multivariate statistical analysis. All above tests were done using the Statistica 10.0 software package (StatSoft, 2006). Discriminant analysis with a forward selection procedure was applied to test which measured features were the most affected in each solution and which discriminated among the NI, B1, and B2 treatments the best (CVA, Canoco 5 package; ter Braak and Šmilauer, 2012).

## Results

### Differences in the Level of Salt Tolerance of Crops

The analysis of the growth parameters of plants grown in different concentrations of NaCl allowed for the differentiation of the tested crops in terms of tolerance to salinity and indicated their significant species diversity in this respect.

**FIGURE 2:**
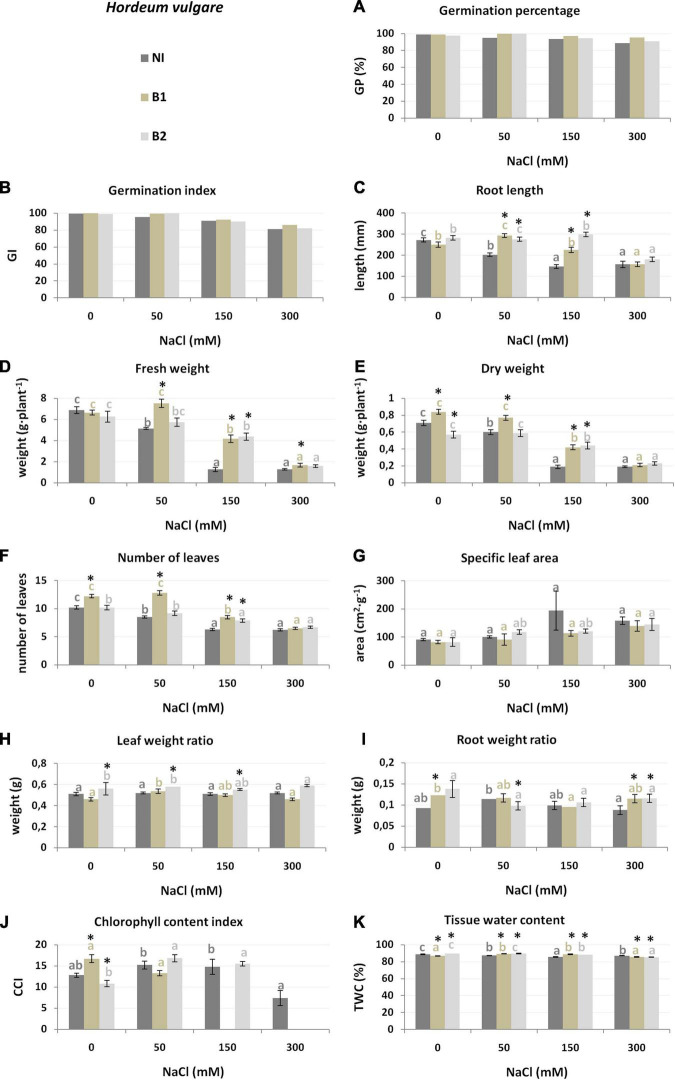
Growth parameters [germination percentage **(A)**, germination index **(B)**, root length **(C)**, fresh weight **(D)**, dry weight **(E)**, number of leaves **(F)**, specific leaf area **(G)**, leaf weight ratio **(H)**, root weight ratio **(I)**, chlorophyll content index **(J)** and tissue water content **(K)** of *H. vulgare* non-inoculated (NI), inoculated with *P. stutzeri* ISE12 (B1) and *K. marisflavi* CSE9 (B2) grown in substrates with different NaCl concentrations (0, 50, 150, and 300 mM NaCl). Significant differences (*p* < 0.05, one-way ANOVA with Tukey’s post hoc comparisons) between treatments (NI, control, non-inoculated; B1, inoculated with *P. stutzeri* ISE12; and B2, inoculated with *K. marisflavi* CSE9) at each NaCl concentration are denoted by different marks (*); differences between values for the investigated growth parameters obtained for each treatment at tested NaCl concentrations (0, 50, 150, and 300 mM) are marked with different letters (including a suitable font for each experiment: dark gray for control: non-inoculated; beige for B1; and light gray for B2) one-way ANOVA with Tukey’s post hoc comparisons]. The mean ± standard error are presented (*n* = 10–15). Data for analysis are given in Supplementary Table 2.

**FIGURE 3:**
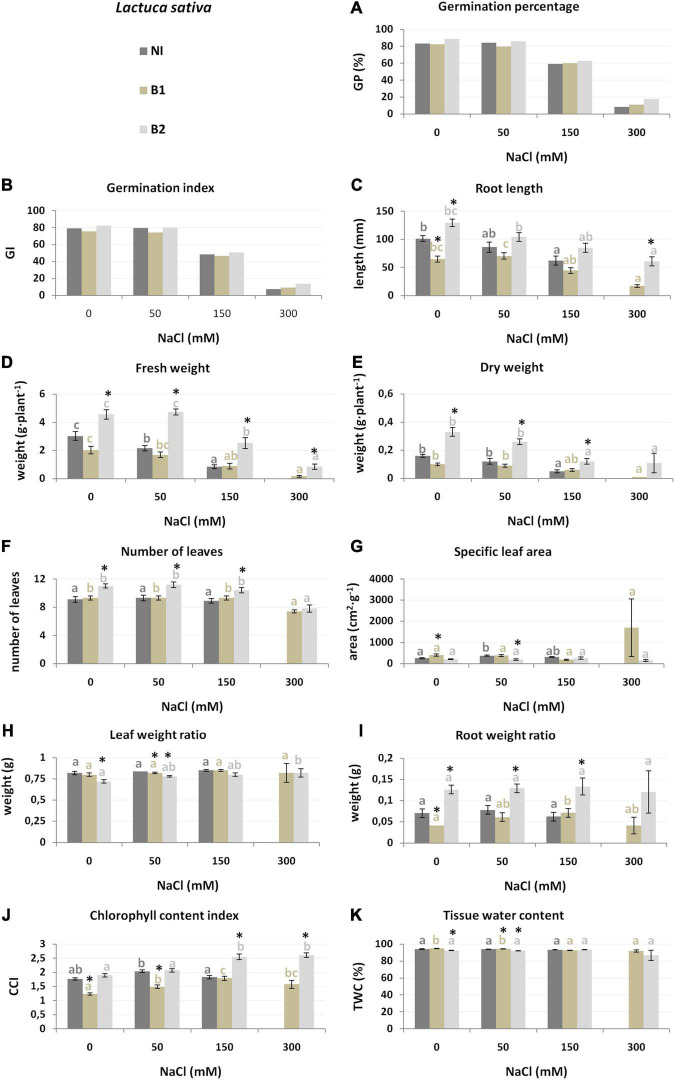
Growth parameters [germination percentage **(A)**, germination index **(B)**, root length **(C)**, fresh weight **(D)**, dry weight **(E)**, number of leaves **(F)**, specific leaf area **(G)**, leaf weight ratio **(H)**, root weight ratio **(I)**, chlorophyll content index **(J)** and tissue water content **(K)** of *L. sativa* non-inoculated (NI), inoculated with *P. stutzeri* ISE-12 (B1) and *K. marisflavi* CSE9 (B2) grown in substrates with different NaCl concentrations (0, 50, 150, and 300 mM NaCl). Significant differences (*p* < 0.05, one-way ANOVA with Tukey’s post hoc comparisons) between treatments (NI, control, non-inoculated; B1, inoculated with *P. stutzeri* ISE12; and inoculated with B2, *K. marisflavi* CSE9) at each NaCl concentration are denoted by different marks (*); differences between values for the investigated growth parameters obtained for each treatment at tested NaCl concentrations (0, 50, 150, and 300 mM) are marked with different letters (including a suitable font for each experiment: dark gray for control: non-inoculated; beige for B1; and light gray for B2) one-way ANOVA with Tukey’s post hoc comparisons]. The mean ± standard error are presented (*n* = 10–15). Data for analysis are given in Supplementary Table 3.

**FIGURE 4:**
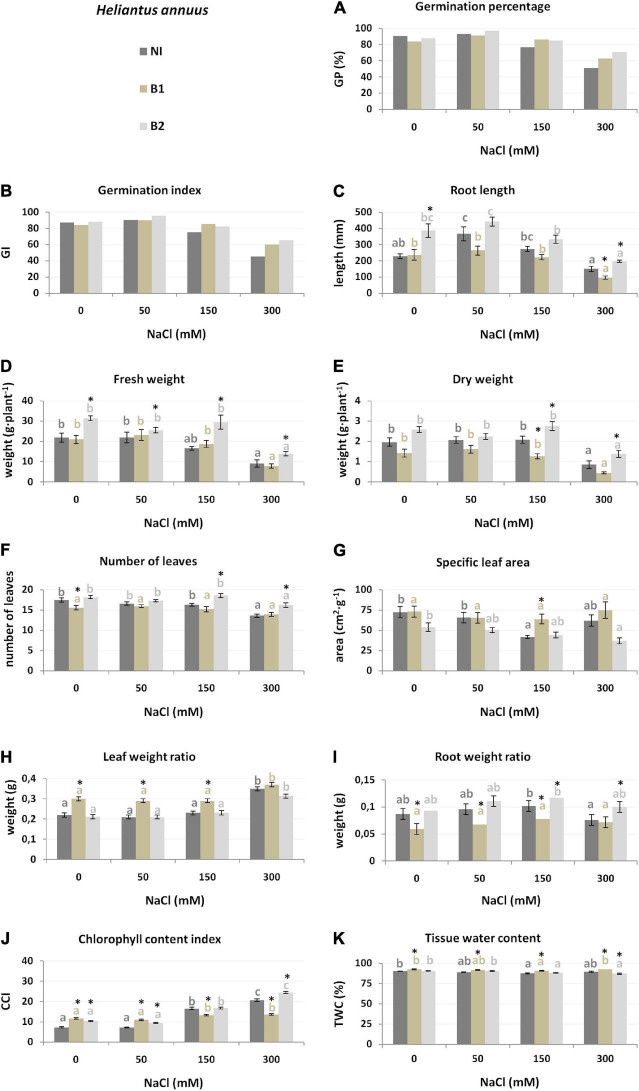
Growth parameters [germination percentage **(A)**, germination index **(B)**, root length **(C)**, fresh weight **(D)**, dry weight **(E)**, number of leaves **(F)**, specific leaf area **(G)**, leaf weight ratio **(H)**, root weight ratio **(I)**, chlorophyll content index **(J)** and tissue water content **(K)** of *H. annuus* non-inoculated (NI), inoculated with *P. stutzeri* ISE-12 (B1) and *K. marisflavi* CSE9 (B2) grown in substrates with different NaCl concentrations (0, 50, 150, and 300 mM NaCl). Significant differences (*p* < 0.05, one-way ANOVA with Tukey’s post hoc comparisons) between treatments (NI, control, non-inoculated; B1, inoculated with *P. stutzeri* ISE12; and inoculated with B2, *K. marisflavi* CSE9) at each NaCl concentration are denoted by different marks (*); differences between values for the investigated growth parameters obtained for each treatment at tested NaCl concentrations (0, 50, 150, and 300 mM) are marked with different letters (including a suitable font for each experiment: dark gray for control: non-inoculated; beige for B1; and light gray for B2) one-way ANOVA with Tukey’s post hoc comparisons]. The mean ± standard error are presented (*n* = 10–15). Data for analysis are given in Supplementary Table 4.

Substrates with increasing salinity (0, 50, 150, 300 mM NaCl) were used for crop cultivation to determine the most significantly affected plant traits (Table 1 and Figures 2–4). *H. vulgare* plants grown under salt condition were smaller than those grown in the control variant (0 mM NaCl): W_f_ (was lower 25.2% in 50 mM compared to the control to 81.7% in 150 and 300 mM), W_d_ (from 15.5 to 73.2%, respectively), NoL (16.7–39.2%), and RL (25.7% to above 42.4%). The TWC was 1.7–3.4% lower under each salt treatment than under the control conditions (Supplementary Tables 1, 2). The germination parameters GP and GI decreased only at the highest salinity (300 mM) (Figure 2).

**TABLE 1:** Two-way ANOVA results of endophytic bacteria (EB) inoculation (NI, non-inoculated; B1, inoculated with: *P. stutzeri* ISE12; and B2, *K. marisflavi* CSE9) and NaCl effects on growth parameters of *Hordeum vulgare, Lactuca sativa*, and *Helianthus annuus*.

ANOVA main effects										

	Parameter	NoL (n)	RL (mm)	Wf (g⋅plant^–1^)	Wd (g⋅plant^–1^)	SLA (cm^2^⋅g^–1^)	LWR (g⋅g^–1^)	RWR (g⋅g^–1^)	TWC (%)	CCI
*Hordeum vulgare*	EB	*p* < 0.0001	*p* < 0.0001	*p* < 0.0001	*p* < 0.0001	ns	ns	*p* < 0.05	*p* < 0.0001	nc
	NaCl	*p* < 0.0001	*p* < 0.0001	*p* < 0.0001	*p* < 0.0001	*p* < 0.01	*p* < 0.0001	ns	*p* < 0.0001	nc
	EBxNaCl	*p* < 0.0001	*p* < 0.0001	*p* < 0.0001	*p* < 0.0001	ns	*p* < 0.0001	*p* < 0.05	*p* < 0.0001	nc
	
EB effect	NI	7.8^a^	194.8^a^	3.64^a^	0.422^a^	135.9^a^	0.515^a^	0.098^a^	87.25^a^	nc
	B1	**10.0^c^**	**231.5^b^**	**5.01^c^**	**0.567^b^**	106.3^a^	**0.489^b^**	**0.113^b^**	87.76^a^	nc
	B2	**8.5^b^**	**259.0^c^**	**4.49^b^**	0.452^a^	116.4^a^	**0.481^b^**	**0.115^c^**	**88.31^b^**	nc

*Lactuca sativa*	EB	*p* < 0.001	*p* < 0.0001	*p* < 0.0001	*p* < 0.0001	*p* < 0.01	*p* < 0.001	*p* < 0.0001	*p* < 0.0001	*p* < 0.0001
	NaCl	*p* < 0.001	*p* < 0.0001	*p* < 0.0001	*p* < 0.0001	ns	*p* < 0.001	*p* < 0.01	*p* < 0.0001	*p* < 0.0001
	EBxNaCl	*p* < 0.01	*p* < 0.01	*p* < 0.0001	*p* < 0.01	*p* < 0.001	*p* < 0.001	ns	*p* < 0.0001	*p* < 0.0001
	
EB effect	NI	6.6^a^	62.45^a^	1.51^a^	0.087^a^	237.2^a^	0.606^a^	0.051^a^	70.4^a^	1.74^b^
	B1	**9.1^b^**	58.36^a^	1.34^a^	0.081^a^	**522.6^b^**	**0.819^c^**	0.055^a^	**93.8^b^**	1.48^a^
	B2	**10.4^c^**	**100.52^b^**	**3.25^b^**	**0.222^b^**	202.2^a^	**0.744^b^**	**0.128^b^**	**91.3^b^**	**2.21^c^**

*Helianthus annuus*	EB	*p* < 0.0001	*p* < 0.0001	*p* < 0.0001	*p* < 0.0001	*p* < 0.0001	*p* < 0.0001	*p* < 0.0001	*p* < 0.0001	*p* < 0.001
	NaCl	*p* < 0.03	*p* < 0.0001	*p* < 0.0001	*p* < 0.0001	*p* < 0.01	*p* < 0.0001	*p* < 0.001	*p* < 0.0001	*p* < 0.001
	EBxNaCl	*p* < 0.0001	ns	*p* < 0.03	ns	ns	*p* < 0.0001	ns	*p* < 0.001	*p* < 0.001
	
EB effect	NI	16.1^a^	259.2^b^	17.41^a^	1.78^a^	60.42^b^	0.247^a^	0.091^b^	89.3^a^	13.1^a^
	B1	15.2^a^	206.2^a^	17.73^a^	1.20^b^	**69.20^b^**	**0.310^b^**	0.070^a^	**92.1^b^**	12.4^a^
	B2	15.6^a^	**315.1^c^**	**25.12^b^**	1.89^a^	46.26^a^	**0.296^b^**	**0.107^c^**	89.3^a^	**15.4^b^**

*Row data for analysis are given in Supplementary Tables 2–4. NoL, number of leaves; RL, root length; W_f_, fresh weight; W_d_, dry weight; SLA, specific leaf area; LWR, leaf weight ratio; RWR, root weight ratio; TWC, tissue water content; CCI, chlorophyll content index; ns, not significant; and nc, not compared. Mean value obtained for all salt concentrations are presented. Significant differences between means in each variant are marked by different letters. Significant positive effects of inoculation are marked in bold.*

In general, the plant genotypes from the family *Asteraceae* (*L. sativa* and *H. annuus*) were similarly affected by salt stress. However, *L. sativa* was more sensitive (non-inoculated plants did not grow in 300 mM NaCl) (Figure 3). *L. sativa* cultivated in substrate supplemented with 150 and 300 mM NaCl had a lower germination ability, as expressed by GP and GI (Figure 3). The growth parameters were also reduced under salt treatment: W_f_ (28.7% lower than the control at 50 mM, 72.2% at 150 mM), W_d_ (25.0 and 68.7%, respectively), and RL (15.1 and 38.8%) (Figure 3 and Supplementary Table 3).

Slightly different strategies to cope with salt stress were demonstrated by *H. annuus*. Similarly, GP and GI decreased under high salinity (150 and 300 mM) (Figure 4), along with W_f_ (24.2% lower compared to the control at 150 mM and 58.4% lower at 300 mM), W_d_ (56.8% at 300 mM), and NoL (22.1% at 300 mM) (Supplementary Table 4). However, RL and RWR first increased significantly at 50 and 150 mM as an adaptation to water deficit and then decreased as a result of salt stress at 300 mM, but only by 34.1% (RL) and 12.6% (RWR). Thus, adaptation to cope with the highest salinity (300 mM NaCl) increased the LWR and CCI (Supplementary Table 4).

In summary, the effect of salinity on plant growth revealed the following order of salt tolerance levels in the investigated crops: *H. annuus* > *H. vulgare* > *L. sativa*.

### Effect of Bacterial Inoculation on Plant Growth Parameters

The differences between the two tested strains (*P. stutzeri* ISE12—B1 and *K. marisflavi* CSE9—B2) were determined on the basis of their effect on the increase of plant growth parameters and more universal to all investigated crops strain was selected. The results of two-way ANOVA demonstrated that both investigated bacterial strains significantly affected most of the investigated plant traits (Table 1). In the case of *H. vulgare*, B1 stimulated NoL development, W_f_ and W_d_, whereas B2 was responsible for better root development (RL and RWR), and water accumulation (TWC). A detailed comparison of salt treatments revealed that bioaugmentation of *H. vulgare* with both investigated strains had a positive effect on plants cultivated in 50 and 150 mM NaCl, as seen for the following parameters: GP, GI, NoL, RL, W_f_, W_d_, LWR, and TWC (Figure 2 and Supplementary Table 2). Under a lower salinity of 50 mM, inoculation of *H. vulgare* with B1 resulted in higher NoL and biomass accumulation (W_f_ and W_d_) compared to that with B2 (Figure 2 and Supplementary Table 2). At 150 mM, both strains stimulated NoL, Wf, Wd, and TWC, but strain B2 affected root growth more strongly (higher RL).

Both species from the *Asteraceae* family (*L. sativa* and *H. annuus*) were more susceptible to B2 inoculation (Table 1). In general, inoculation of *L. sativa* with B2 increased most of the growth parameters. Detailed analysis of the salt treatments demonstrated that under the highest salinity (300 mM), strain B2 stimulated RL, W_f_, W_d_, and CCI, whereas under lower salinity (50 and 150 mM), NoL and RWR were stimulated (Figure 3 and Supplementary Table 3). The role of B1 in growth stimulation was much smaller than that of B2. Moreover, some traits were negatively affected by this strain, e.g., RL and CCI (Figure 3 and Supplementary Table 3).

The growth of *H. annuus* was also better stimulated by B2 (Table 1), resulting in stronger root development (RL and RWR), photosynthesis ability (CCI and LWR), and fresh biomass accumulation (W_f_), whereas strain B1 positively affected mainly water accumulation (TWC) and photosynthesis ability (LWR). In general, B2-inoculated plants were characterized by better seed germination (GP and GI) (Figure 4). In all salinity treatments, these plants had better developed roots (RL and RWR), higher biomass (W_f_ and W_d_), and a 50 mM higher chlorophyll concentration in the leaves (CCI) (Figure 4 and Supplementary Table 4). Conversely, decreases in growth parameters, e.g., Wd, RWR, and CCI, were observed after inoculation with B1. The results clearly show a lack of compatibility between *H. annuus* and *P. stutzeri* ISE12; moreover, an adverse influence of that strain was observed.

**FIGURE 5:**
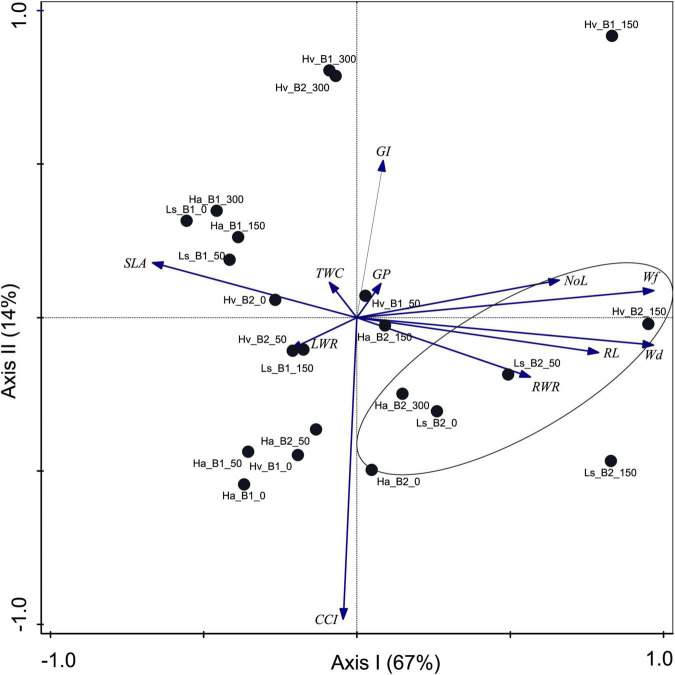
Biplot of the principal component analysis of germination and growth parameters (GP, germination percentage; GI, germination index; NoL, number of leaves; RL, root length; W_f_, fresh weight; W_d_, dry weight; SLA, specific leaf area; LWR, leaf weight ratio; RWR, root weight ratio; TWC, tissue water content; CCI, chlorophyll content index) obtained for *H. vulgare* (Hv), *L. sativa* (Ls), and *H. annuus* (Ha) inoculated with *P. stutzeri* ISE12 (B1) and K. marisflavi CSE9 (B2) cultivated in substrates enriched with different salinities (0, 50, 150, and 300 mM NaCl). Differences between the values for the parameters obtained for the control (non-inoculated) and inoculated variants were included. B2 treatments with the best relative growth are marked with a circle.

The PCA results presented in Figure 5 show the percentage differences between plant traits for inoculated and control (NI) treatments, which included three plant species (*H. vulgare*, *L. sativa*, *H. annuus*) and four different levels of salinity (0, 50, 150, 300 mM NaCl). Positive correlations were found between *L. sativa* (all investigated variants: Ls_B2_0, Ls_B2_50, Ls_B2_150) and *H. annuus* (three of the four variants: Ha_B2_0, Ha_B2_150, Ha_B2_300) inoculated with B2 under most salinity treatments among the NoL, W_f_, W_d_, RL, and RWR traits. Bioaugmentation of plants from this family with B1 was associated only with SLA, TWC, and LWR. Inoculation of all investigated genotypes with B2 stimulated growth parameters according to the following trend: *L. sativa* > *H. annuus* > *H. vulgare* (Figure 5). Strain B1 revealed the opposite trend and had no evident effect.

### Plant Growth Parameters Were Susceptible to Changes in Response to Salinity and Inoculation

Based on the obtained results, an analysis was carried out, which allowed for the selection of plant growth parameters most sensitive to increasing salinity and bacterial inoculation. The results of the discriminant analysis and forward selection procedure revealed that salinity modified the effect of EB in a quantitative way. In the case of *H. vulgare*, the most critical feature for discrimination between the NI, B1, and B2 groups in all salinity treatments was the water content in plants (TWC), which explained 28–14% of the total variation (Table 2). Moreover, under 0 mM NaCl, the most affected plant parameters were LWR (explaining 48.2% of the trait variance between the NI, B1, and B2 groups) and CCI (7.5%) (Supplementary Figure 1). At 50 mM, in addition to TWC, significant group separation was found for NoL (31.4%), and in 150 mM, the same was found for RL (40.5%).

**TABLE 2:** Results of discriminant analysis (CVA) between NI, B1, and B2 treatments under different NaCl concentrations—percentage of the variance explained between groups for each growth parameter.

	*H. vulgare* mM NaCl				*L. sativa* mM NaCl				*H. annuus* mM NaCl			
			
	0	50	150	300	0	50	150	300*	0	50	150	300
NoL	0.4	**31.4**	1.5	2.7	**9.3**	0.4	1.7	nc	7.7	7.9	2.0	**14.1**
RL	<0.1	3.2	**40.5**	1.9	0.5	<0.1	**15.7**	**62.9**	3.4	0.5	0.3	0.2
Wf	1.1	3.4	0.2	7.8	7.9	2.4	1.7	4.8	**13.4**	0.5	**8.5**	1.3
Wd	0.6	2.1	3.9	3.7	2.2	**44.9**	**27.3**	13.7	0.2	2.1	9.4	0.8
SLA	5.1	2.2	1.0	1.6	<0.1	**39.8**	8.3	0.6	3.7	2.4	<0.1	0.6
LWR	**48.2**	0.8	8.8	0.4	2.2	1.5	3.1	1.8	**24.4**	2.9	5.3	1.0
RWR	1.2	7.8	<0.1	8.5	**37.9**	1.3	3.4	0.6	0.4	**24.0**	**30.7**	6.8
TWC	**28.0**	**25.1**	**14.6**	**17.0**	2.2	0.7	0.7	7.5	1.4	10.6	**18.1**	9.9
CCI	**7.5**	0.8	nc	nc	**15.6**	0.6	1.7	1.1	**22.4**	**9.1**	<0.1	**34.9**

Figures associated with presented data are given in Supplementary Figures 1–3. NoL, number of leaves; RL, root length; W_f_, fresh weight; W_d_, dry weight; SLA, specific leaf area; LWR, leaf weight ratio; RWR, root weight ratio; TWC, tissue water content; CCI, chlorophyll content index; and nc, not compared. Plants in non-inoculated control at 300 mM did not survive and are denoted by marks *. Significant factors (p < 0.01) are underlined in bold.

The *L. sativa* analysis demonstrated that the most essential features for discrimination between groups were at 0 mM NaCl root development, indicated as RWR (explaining 38% of the variation), chlorophyll index (CCI, 16%), and number of leaves (NoL, 9%) (Table 2). At 50 mM NaCl, the most affected traits were W_d_ (explaining 48.2% of the variance between the NI, B1, and B2 groups) and SLA, whereas under 150 mM, W_d_ (27%) and RL (16%) were the most affected, and under 300 mM onlyRL was the most affected (63%, NI plants died) (Supplementary Figure 2).

The most essential trait for discrimination between the NI, B1, and B2 treatments for *H. annuus* was the chlorophyll index (CCI, explaining 9–35% of trait variance) across all NaCl concentrations, except for 150 mM (Table 2). Moreover, under 0 mM NaCl, the critical features for discrimination between the NI, B1, and B2 treatments were LWR (explaining 24% of the variation) and W_f_, while under 50 mM, root development, expressed as RWR (24%), under 150 mM, RWR (31%) and TWC (18%) and under 300 mM NaCl, fresh weight (W_f_, 8%) and NoL (14%) (Supplementary Figure 3).

This analysis allowed us to designate chlorophyll concentration (CCI), the development of leaves (NoL, LWR, SLA), water storage (TWC), root development (RL, RWR), and biomass accumulation (W_f_, W_d_) as crucial traits affected by salinity and bacterial inoculation. However, the effects reported here are species specific.

## Discussion

The adverse effect of salinity on the germination and growth of plants has been confirmed for many crop species, e.g., *Zea mays* (maize), *Lycopersicon esculentum* (tomato), *Oryza sativa* (rice), *Glycine max* (soybean) *Beta vulgaris* (sugar beet), *Brassica oleracea capitata* (cabbage), *Amaranthus paniculatus* (amaranth), and *Brassica campestris* (pak-choi) (Jamil et al., 2006; Doganlar et al., 2010; Turan et al., 2010; Vibhuti et al., 2015); *Brassica napus* and *Beta vulgaris* (Piernik et al., 2017; Khan et al., 2019a,^2020^; Szymańska et al., 2019, 2020; Masmoudi et al., 2021); and *Sorghum bicolor* (Rajabi Dehnavi et al., 2020). Our present research confirmed the negative effect of salinity on *H. vulgare*, *L. sativa*, and *H. annuus* and additionally showed that this effect is conspicuously associated with a reduction in the germination percentage and index, root length, and fresh and dry weights. The assessment of the three-way interaction, saline substrate—crop—endophyte, allowed us to determine the key factors responsible for compatibility between bacterial species and plant genotypes. Since the inoculation of crops with the most efficient plant growth-promoting bacteria (PGPB) is one of the relevant strategies used in sustainable agriculture in salt-affected areas (Kumar et al., 2020), searching for new compatible microorganisms appropriately matched to the plant host and environmental conditions is crucial. *P. stutzeri* is a well-described bacterium and is characterized as a PGPB due to several of its beneficial metabolic properties, e.g., IAA and siderophore synthesis, nitrogen fixation, antifungal activity, and 1-aminocyclopropane-1-carboxylate (ACC) deaminase synthesis (Patel and Patel, 2014; Han et al., 2015; Pham et al., 2017; Lami et al., 2020; Kumar et al., 2021). However, there is a lack of research considering the application potential of *K. marisflavi*. We previously initiated the first studies on the use of this bacteria with two crops (*Beta vulgaris* and *Brassica napus*) (Piernik et al., 2017; Szymańska et al., 2019, 2020) and expanded the range of crops tested in this work.

### Salt Stress Tolerance of Plant Species

Determination of plant germination capacity under high salinity conditions is a crucial parameter for assessing plant tolerance to salt stress (Li et al., 2020). Salinity adversely affects the germination stage by limiting water availability and changing stored reserve mobilization and structural protein organization (Demir and Mavi, 2008; Nawaz et al., 2010). The negative effect on germination can be genotype- or cultivar-specific (Al-Ashkar et al., 2020; Li et al., 2020; Kadioǧlu, 2021). In the present study, we observed significant differences in salinity between the three investigated crop genotypes. Li et al. (2020) studied the different seed germination stage parameters (such as germination rate, germination index, germination energy, germination vigor index, and water content) of 552 sunflower germplasms with different levels of salt stress tolerance. The authors found that the germination index and the germination vigor index were the most reliable traits and were strongly associated with salt tolerance. In our work, increasing the concentration of NaCl in substrates inhibited seed germination; however, the effect was species-specific. The effect of salinity on the germination of *H. annuus* and *L. sativa* was particularly evident at higher salinities (150 and 300 mM NaCl). However, across all tested plant species, we did not observe decreased germination at 50 mM NaCl. This is because a low concentration of Na^+^ has a positive effect on plant growth, since Na^+^ participates in osmotic and nutritional mechanisms. This effect was also observed in our studies on red beet and canola (Szymańska et al., 2019, 2020), as well as in experiments with red beet, wheat and *Zygophyllum xanthoxylum* described by Wu et al. (2013). *H. annuus* and *H. vulgare* are crops with the highest levels of salt stress tolerance (Garthwaite et al., 2005; Li et al., 2020; Vasilakoglou et al., 2021), which we confirmed in the experiments presented in this work. In contrast, we observed relatively low tolerance of *L. sativa* to salinity, especially at the highest concentration of NaCl (300 mM), where we observed complete inhibition of germination and plant growth. Moreover, gradual plant death was observed over several weeks during the experiment. Ludwiczak et al. (2021) showed that osmotic stress rather than ionic effects is responsible for this process.

### Compatibility Between Plant Growth-Promoting Bacterial Strains and Plant Genotypes

The compatibility between selected strains was plant specific. Both endophytes (*P. stutzeri* ISE12 and *K. marisflavi* CSE9) increased *H. vulgare* adaptation to salt stress, which was confirmed by the growth parameters, especially under supplementation with 50 and 150 mM NaCl. Similarly, a positive effect of the same strains was observed in our earlier studies with *B. napus* and *B. vulgaris* (Szymańska et al., 2019, 2020).

Our results suggest that crops from the same family growing under similar conditions (e.g., substrate, temperature, light) can be compatible with the same microorganisms. Our results show that *H. annuus* and *L. sativa* (family Asteraceae) inoculated with *K. marisflavi* CSE9 showed increases in the same growth parameters (germination percentage and index, number of leaves, fresh weight, root length, root weight ratio, chlorophyll content index). However, the positive effect of inoculation with a specific strain may also be related to the specific metabolic activities of bacteria used for inoculation. *P. stutzeri* ISE12 possesses a wider range of metabolic properties (siderophores and IAA synthesis, cellulolytic activity, and presence of *nif*H) than *K. marisflavi* CSE9 (siderophores and IAA synthesis) (Szymańska et al., 2020). Moreover, although both strains showed high salinity tolerance, we confirmed that *K. marisflavi* CSE9 had higher potential (Szymańska et al., 2020). Both strains had IAA synthesis ability, and multifunctional phytohormones play crucial roles in plant growth and development promotion as well as stress resistance (Numan et al., 2018; Khan et al., 2019b; Saleem et al., 2021). Egamberdieva (2009) observed higher crop productivity after the inoculation of wheat with IAA-producing strains under salinity stress than without salt (2008). Similarly, the mitigation of salt stress and stimulation of plant growth was noted for cotton, and the authors suggested that IAA synthesized by bacteria can be an extremely important factor in the salt stress tolerance of plants (Egamberdieva et al., 2015).

### Crucial Traits Affected by Salinity and Inoculation

Based on the results obtained in the study, we also determined universal parameters that are the most affected by inoculation under salt stress. However, we recognized that the inoculation effect was dependent on the salinity level and was species specific. In the case of *H. vulgare*, we identified TWC as the most affected trait in all salt treatments. In general, bacteria increased the water content in plant tissues at 50 and 150 mM NaCl, but at 300 mM, the water content significantly decreased. This trait was also independently affected by salinity level. TWC decreased together with increased salinity. This result is in line with those by Ludwiczak et al. (2021), who identified the TWC of *H. vulgare* and *A. sativa* as one of the most affected traits under 150 mM NaCl. However, Garthwaite et al. (2005) investigated the salt tolerance of eight wild *Hordeum* species (including *H. bogdanii*, *H. intercedens*, *H. jubatum*, *H. lechleri*, *H. marinum*, *H. murinum*, *H. patagonicum*, and *H. secalinum*) and cultivated barley (*H. vulgare*) and observed the smallest reduction in this parameter for *H. vulgare* compared to the wild *Hordeum* species. On the other hand, stimulation of tissue water storage after inoculation by endophytes was also reported for draught stressed maize by Naveed et al. (2014) and lettuce by Molina-Montenegro et al. (2016). Additional crucial traits of *H. vulgare* were the number of leaves (NoL) under 50 mM NaCl and RL under 150 mM NaCl, which were the most stimulated by B2 bacteria.

The RL trait was also crucial for discriminating between *L. sativa* treatments and was stimulated by B2 bacteria, especially at 150 and 300 mM NaCl. The reduction in root length under salt stress may result from the direct contact of the roots with an unfavorable abiotic factor (Jamil et al., 2006; Vibhuti et al., 2015; Peng-cheng et al., 2021). The stimulation of root growth may be a solution for the improvement of water absorption under salt stress (Piernik et al., 2017).

Interestingly, in the case of lettuce, TWC was not a significant factor for discriminating the investigated treatments. Al-Maskri et al. (2010) noted a negative effect of salinity on several *L. sativa* growth parameters (including the number of leaves, plant fresh weight, shoot fresh and dry weight, shoot dry matter percentage, root fresh and dry weight, root dry weight percentage, leaf area and leaf area index), and similar to our work, they did not observe adverse effects on the root and shoot water content percentages. The opposite results were obtained by Ahmed et al. (2019), who observed a gradual reduction in the water content in *L. sativa* roots and shoots with increasing NaCl concentration (2019). Many differences in the growth parameters of *L. sativa* grown under salt stress have been observed by various researchers (e.g., Bar-Yosef et al., 2005; Ünlükara et al., 2008; Kurunc, 2021). Presumably, the varieties of *L. sativa* are characterized by large differences in salinity tolerance, and plant responses may be related to other morphological and physiological parameters.

Not only the root length but also the root weight ratio (RWR) was crucial for the discrimination between NI, B1, and B2 treatments and was positively stimulated by B2 bacteria, especially in the case of *L. sativa* and *H. annuus*. This result suggests changes in the plant growth strategy and a higher investment in root development with endophyte inoculation (Reynolds and D’Antonio, 1996).

For *L. sativa* and *H. annuus*, biomass accumulation was of crucial importance in the context of salinity and endophytic bacteria interactions. The dry weight (W_d_) showed that the best salt levels were 50 and 150 mM NaCl for lettuce. This trait was significantly stimulated by B2 bacteria. The fresh weight (W_f_) showed that the best salt levels were 0 and 150 mM for sunflower and was also stimulated by B2 (*K. marisflavi*).

The most interesting result is the impact of the investigated endophytes on the chlorophyll content, expressed here as CCI. Inoculation with B1 (*P. stutzeri*) increased the CCI of *H. vulgare*, whereas inoculation with B2 increased the CCI of *L. sativa* under the 0 mM control conditions. Moreover, the CCI parameter was crucial for discriminating across the investigated treatments of *H. annuus* at 0, 50, and 300 mM, where B2 bacteria were responsible for higher values. An increase in chlorophyll content after inoculation with endophytic bacteria has also been observed by other researchers (e.g., Zhao et al., 2015; Yuan et al., 2018; Tharek et al., 2021). In our previous research, we found the same effect of *P. stutzeri* on beetroot (Piernik et al., 2017; Szymańska et al., 2020). A higher CCI may result in a higher efficiency of photosynthesis under environmental stress (Zhao et al., 2020).

## Conclusion

In conclusion, our results indicated that salinity reduced crop productivity, but the effect was species specific: *H. annuus* > *H. vulgare* > *L. sativa*. For the most crucial traits affected by endophyte inoculation under salt stress, we found that chlorophyll concentration (CCI), the development of leaves (mostly NoL), water storage (TWC), root development (RL, RWR), and biomass accumulation (W_f_, W_d_) were the most important. However, all these effects are species specific. We can confidently conclude that *K. marisflavi* is a unique and universal strain that promoted the growth of all tested genotypes, while *P. stutzeri* ISE12 is species specific and requires compatibility testing with a specific host plant before application in the field.

## Data Availability Statement

The original contributions presented in the study are included in the article/Supplementary Material, further inquiries can be directed to the corresponding author/s.

## Author Contributions

SS performed the pot experiment, plant growth parameters analysis and part of statistical analysis, and wrote the first version of the manuscript. KH determined the first concept of the experiment and participated in the preparation of the manuscript. AP analyzed the data and wrote part of materials and methods as well as results. ML contributed in the laboratory work and performed statistical analysis. All authors contributed to the final version.

## Conflict of Interest

The authors declare that the research was conducted in the absence of any commercial or financial relationships that could be construed as a potential conflict of interest.

## Publisher’s Note

All claims expressed in this article are solely those of the authors and do not necessarily represent those of their affiliated organizations, or those of the publisher, the editors and the reviewers. Any product that may be evaluated in this article, or claim that may be made by its manufacturer, is not guaranteed or endorsed by the publisher.

## Supplementary Material

Supplementary Figure 1 Results of discriminant analysis (CVA) between *Hordeum vulgare* inoculated with *P. stutzeri* ISE12 (B1) and *K. marisflavi* CSE9 (B2) and non-inoculated (NI) variants in different NaCl solutions. Significant parameters that discriminate the best between groups are denoted by stars (*p* < 0.01).

Supplementary Figure 2 Results of discriminant analysis (CVA) between *Lactuca sativa* inoculated with *P. stutzeri* ISE12 (B1) and *K. marisflavi* CSE9 (B2) and non-inoculated (NI) variants in different NaCl solutions. Significant parameters that discriminate the best between groups are denoted by stars (*p* < 0.01). In 300 mM NaCl, only B1 and B2 treatments were compared (NI plants died).

Supplementary Figure 3 Results of discriminant analysis (CVA) between *Helianthus annuus* inoculated with *P. stutzeri* ISE12 (B1) and *K. marisflavi* CSE9 (B2) and non-inoculated (NI) variants in different NaCl solutions. Significant parameters that discriminate the best between groups are denoted by stars (*p* < 0.01).

Supplementary Table 1 Effect of endophytic bacteria inoculation (NI: non-inoculated, B1: inoculated with *P. stutzeri* ISE12, and B2: inoculated with *K. marisflavi* CSE9) on plant tissue water content (TWC) in different NaCl concentrations (0, 50, 150 and 300 mM NaCl). Means ± SE are presented.

Supplementary Table 2 Effect of endophytic bacteria inoculation (NI: non-inoculated, B1: inoculated with *P. stutzeri* ISE12, and B2: inoculated with *K. marisflavi* CSE9) on Hordeum vulgare growth parameters in different NaCl concentrations. Means ± SE are presented.

Supplementary Table 3 Effect of endophytic bacteria inoculation (NI: non-inoculated, B1: inoculated with *P. stutzeri* ISE12, and B2: inoculated with *K. marisflavi* CSE9) on *Lactuca sativa* growth parameters in different NaCl concentrations. Means ± SE are presented.

Supplementary Table 4 Effect of endophytic bacteria inoculation (NI: non-inoculated, B1: inoculated with *P. stutzeri* ISE12, and B2: inoculated with *K. marisflavi* CSE9) on *Helianthus annuus* growth parameters in different NaCl concentrations. Means ± SE are presented.
