# Supplementary figures and images for: Pseudomonas stutzeri and Kushneria marisflavi Alleviate Salinity Stress-Associated Damages in Barley, Lettuce, and Sunflower
Source: Front Microbiol. 2022 Mar 8;13:788893. doi: 10.3389/fmicb.2022.788893 (PMC8957930; doi:10.3389/fmicb.2022.788893)

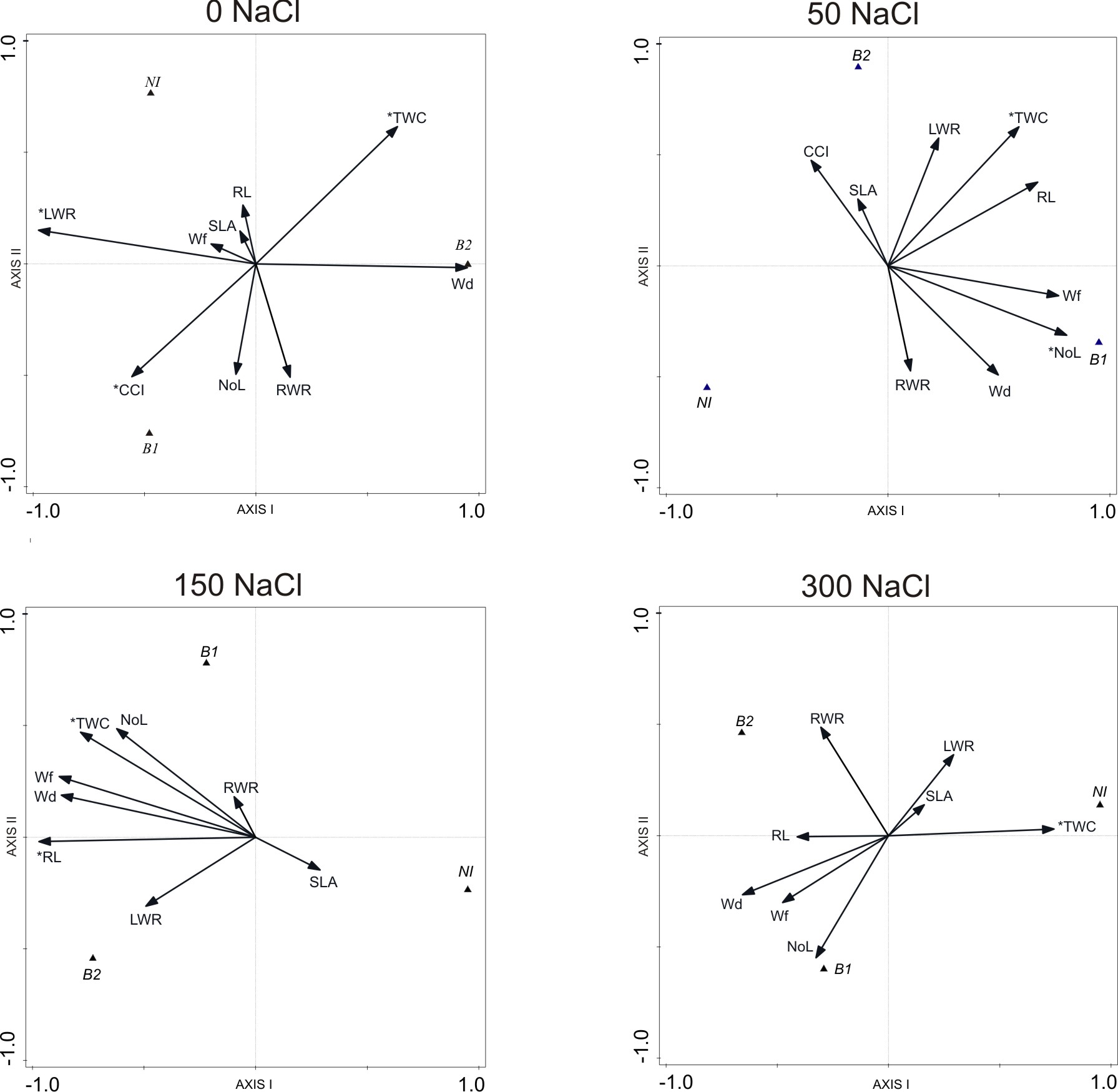

Supplement: Supplementary Figure 1 — Results of discriminant analysis (CVA) between Hordeum vulgare inoculated with P. stutzeri ISE12 (B1) and K. marisflavi CSE9 (B2) and non-inoculated (NI) variants in different NaCl solutions. Significant parameters that discriminate the best between groups are denoted by stars (p < 0.01). [file Image_1.JPEG]

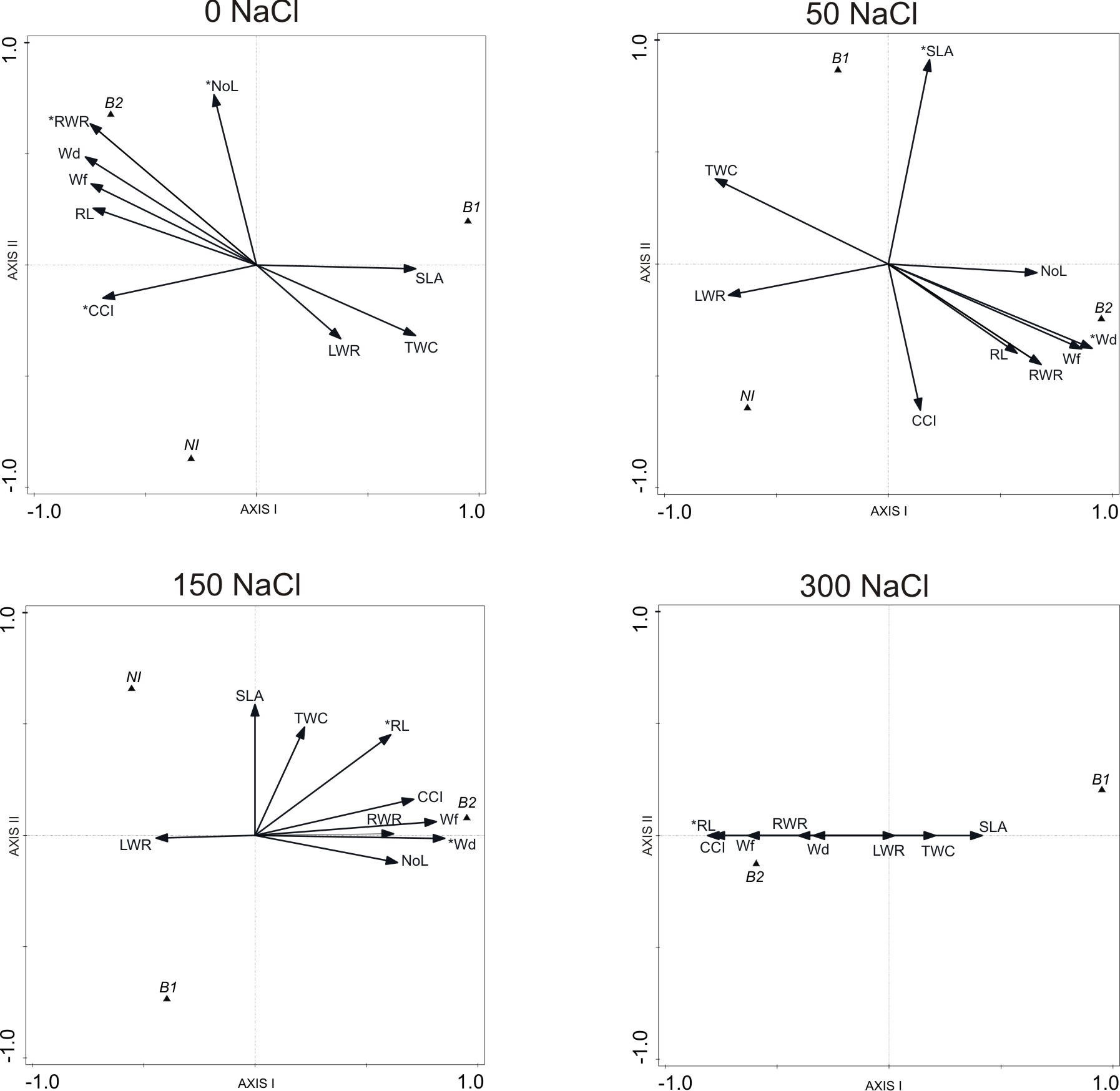

Supplement: Supplementary Figure 2 — Results of discriminant analysis (CVA) between Lactuca sativa inoculated with P. stutzeri ISE12 (B1) and K. marisflavi CSE9 (B2) and non-inoculated (NI) variants in different NaCl solutions. Significant parameters that discriminate the best between groups are denoted by stars (p < 0.01). In 300 mM NaCl, only B1 and B2 treatments were compared (NI plants died). [file Image_2.JPEG]

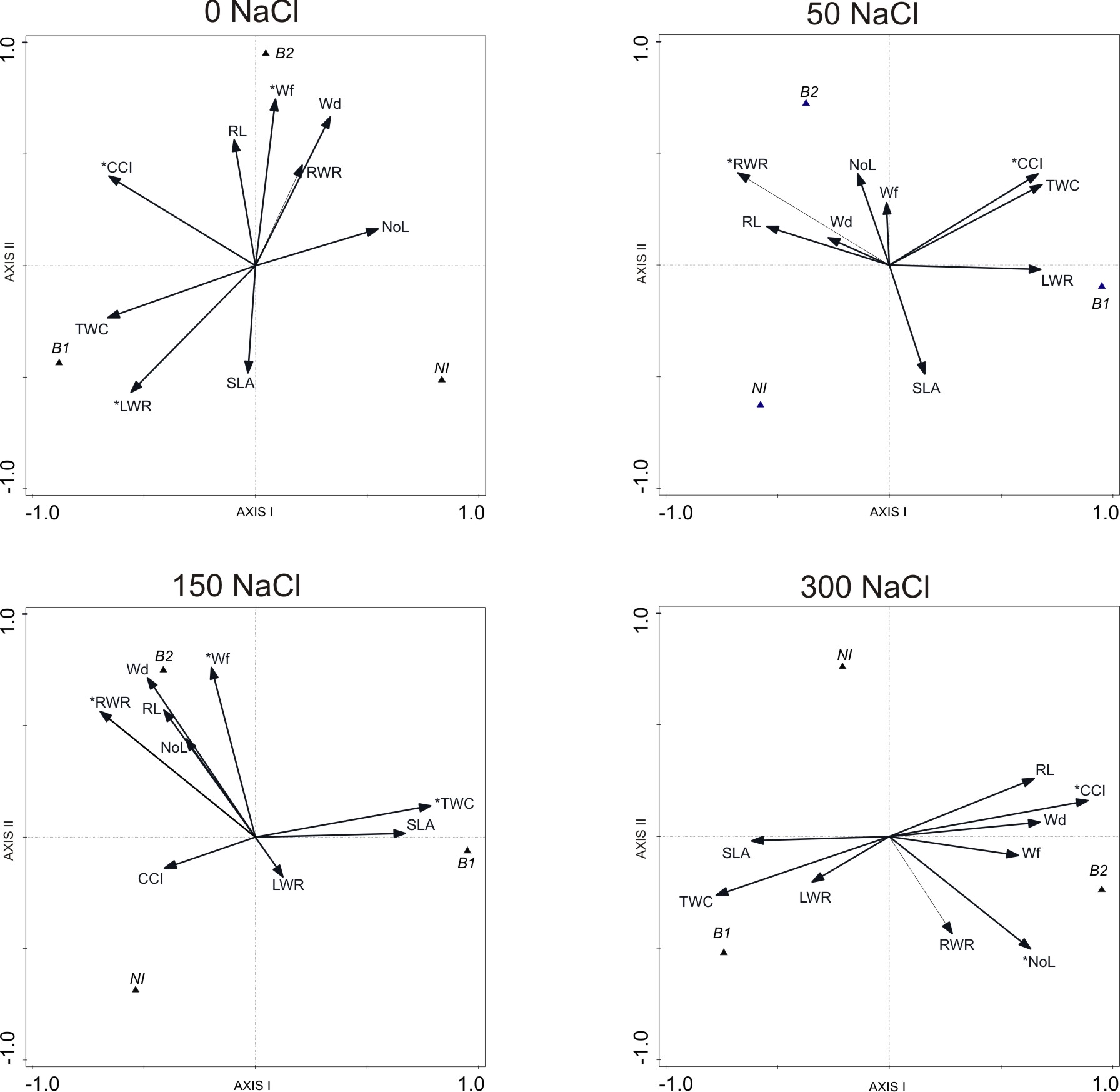

Supplement: Supplementary Figure 3 — Results of discriminant analysis (CVA) between Helianthus annuus inoculated with P. stutzeri ISE12 (B1) and K. marisflavi CSE9 (B2) and non-inoculated (NI) variants in different NaCl solutions. Significant parameters that discriminate the best between groups are denoted by stars (p < 0.01). [file Image_3.JPEG]
