# Supplementary material for: Pseudomonas stutzeri and Kushneria marisflavi Alleviate Salinity Stress-Associated Damages in Barley, Lettuce, and Sunflower
Source: Front Microbiol. 2022 Mar 8;13:788893. doi: 10.3389/fmicb.2022.788893 (PMC8957930; doi:10.3389/fmicb.2022.788893)
Supplement: Supplementary Table 1 — Effect of endophytic bacteria inoculation (NI: non-inoculated, B1: inoculated with P. stutzeri ISE12, and B2: inoculated with K. marisflavi CSE9) on plant tissue water content (TWC) in different NaCl concentrations (0, 50, 150 and 300 mM NaCl). Means ± SE are presented. [file Table_1.docx]

Supplementary Tables

***Pseudomonas stutzeri* and *Kushneria marisflavi* Alleviate Salinity Stress-Associated Damages in Barley, Lettuce, and Sunflower**

**Szymańska Sonia^1*^, Lis Marta^2^, Piernik Agnieszka^2^, Hrynkiewicz Katarzyna^1*^**

**Table 1.** Effect of endophytic bacteria inoculation (NI: non-inoculated, B1: inoculated with *P. stutzeri* ISE12, and B2: inoculated with *K. marisflavi* CSE9) on plant tissue water content (TWC) in different NaCl concentrations (0, 50, 150 and 300 mM NaCl). Means ± SE are presented.

| *Hordeum vulgare* | | | | | |
| --- | --- | --- | --- | --- | --- |
| TWC[%] | mM NaCl | | | | |
|  |  | 0 | 50 | 150 | 300 |
| In total biomass | NI | 88.8 ^b^±0.27 | 87.3 ^a^±0.39 | 85.8 ^a^±0.43 | 87.2 ^b^±0.26 |
|  | B1 | 86.9 ^a^±0.28 | **89.5 ^b^±0.32** | **88.9 ^b^±0.35** | 85.8 ^a^±0.38 |
|  | B2 | **89.7 ^c^±0.16** | **89.7 ^b^±0.24** | **88.4 ^b^±0.15** | 85.5 ^a^±0.37 |
|  |  |  |  |  |  |
| In leaves | NI | 87.1 ^b^±0.20 | 86.5 ^a^±0.28 | 85.5 ^a^±0.27 | 84.8 ^b^±0.56 |
|  | B1 | 84.2 ^a^±0.50 | 87.1 ^ab^±0.35 | 86.7 ^a^±0.44 | 83.5 ^ab^±0.66 |
|  | B2 | 88.2 ^a^±0.16 | **87.9 ^b^±0.34** | 85.4 ^a^±0.39 | 81.9 ^a^±0.69 |
|  |  |  |  |  |  |
| In roots | NI | 90.6 ^a^±0.85 | 86.0 ^a^±1.02 | 63.0 ^a^±4.25 | 89.8 ^a^±0.74 |
|  | B1 | 90.1 ^a^±0.36 | **91.6 ^b^±0.41** | **92.5 ^b^±0.28** | 90.9 ^a^±0.57 |
|  | B2 | 91.3 ^a^±0.29 | **92.1 ^b^±0.21** | **93.0 ^b^±0.16** | 89.8 ^a^±1.16 |

| *Lactuca sativa* | | | | | |
| --- | --- | --- | --- | --- | --- |
| TWC[%] | mM NaCl | | | | |
|  |  | 0 | 50 | 150 | 300 |
| In total biomass | NI | 94.0 ^b^±0.46 | 94.1 ^b^±0.15 | 93.5 ^a^±0.18 | - |
|  | B1 | 95.0 ^b^±0.17 | 94.5 ^b^±0.14 | 92.8 ^a^±0.21 | 92.0 ^a^±1.29 |
|  | B2 | 92.6 ^a^±0.33 | 92.2 ^a^±0.33 | 93.5 ^a^±0.32 | 87.0 ^a^±6.03 |
|  |  |  |  |  |  |
| In leaves | NI | 94.7 ^b^±0.08 | 94.4 ^b^±0.16 | 93.8 ^a^±0.16 | - |
|  | B1 | 95.1 ^b^±0.16 | 94.7 ^b^±0.12 | 93.2 ^a^±0.16 | 93.3 ^a^±0.33 |
|  | B2 | 93.1 ^a^±0.22 | 92.9 ^a^±0.28 | 94.1 ^a^±0.42 | 87.0 ^a^±6.54 |
|  |  |  |  |  |  |
| In roots | NI | 79.5 ^ab^±3.22 | 81.6**^b^**±1.91 | 68.7 ^a^±3.05 | - |
|  | B1 | 72.4 ^a^±2.97 | 56.1 ^a^±4.09 | 69.2 ^a^±3.61 | 93.2 ^a^±2.25 |
|  | B2 | 88.5 ^b^±0.76 | 88.4 ^b^±0.75 | **82.0 ^b^±2.29** | 69.1 ^a^±13.69 |

| *Helianthus annuus* | | | | | |
| --- | --- | --- | --- | --- | --- |
| TWC[%] | mM NaCl | | | | |
|  |  | 0 | 50 | 150 | 300 |
| In total biomass | NI | 90.4 ^a^±0.34 | 89.2 ^a^±0.40 | 87.7 ^a^±0.48 | 89.8 ^b^±0.86 |
|  | B1 | **92.6 ^b^±0.52** | **91.8 ^b^±0.43** | **91.0 ^b^±0.33** | **92.9 ^c^±0.12** |
|  | B2 | 90.7 ^a^±0.33 | 90.5 ^ab^±0.30 | 88.5 ^a^±0.47 | 87.2 ^a^±0.81 |
|  |  |  |  |  |  |
| In leaves | NI | 85.6 ^a^±0.53 | 84.8 ^a^±0.48 | 82.3 ^a^±0.66 | 87.1**^b^**±0.47 |
|  | B1 | 86.4 ^a^±0.69 | **86.9 ^b^±0.57** | **87.0 ^b^±0.55** | 89.6 ^b^±0.29 |
|  | B2 | 85.5 ^a^±0.38 | 86.2 ^ab^±0.26 | 82.9 ^a^±0.57 | 78.9 ^a^±2.40 |
|  |  |  |  |  |  |
| In roots | NI | 92.7 ^a^±0.19 | 92.4 ^a^±0.26 | 92.2 ^a^±0.18 | 91.5 ^b^±0.54 |
|  | B1 | 93.1 ^a^±0.46 | 92.9 ^a^±0.37 | **93.3 ^b^±0.37** | **93.8 ^c^±0.35** |
|  | B2 | 93.1 ^a^±0.37 | **94.0 ^b^±0.26** | 91.8 ^a^±0.21 | 89.0 ^a^±0.42 |

Significant differences between NI, B1 and B2 variants in each NaCl solution are denoted by different letters (one-way ANOVA with Tuckey post-hoc comparison). B1 and B2 significant positive effects are in bold, negative are underlined.

**Table 2.** Effect of endophytic bacteria inoculation (NI: non-inoculated, B1: inoculated with *P. stutzeri* ISE12, and B2: inoculated with *K. marisflavi* CSE9) on *Hordeum vulgare* growth parameters in different NaCl concentrations. Means ± SE are presented.

|  | mMNaCl | | | | |
| --- | --- | --- | --- | --- | --- |
|  |  | 0 | 50 | 150 | 300 |
| NoL |  |  |  |  |  |
| [n] | NI | 10.2^a^±0.3 | 8.5^a^±0.2 | 6.3^a^ ±0.2 | 6.2^a^±0.2 |
|  | B1 | **12.2^b^±0.3** | **12.8^b^±0.4** | **8.5^b^±0.3** | 6.5^a^±0.2 |
|  | B2 | 10.2^a^±0.4 | 9.2^a^±0.4 | **7.9^b^±0.3** | 6.7^a^±0.2 |
| RL |  |  |  |  |  |
| [mm] | NI | 272.6^a^±10.6 | 202.5^a^±8.3 | 147.0^a^±9.3 | 157.1 ^a^±15.3 |
|  | B1 | 250.3^a^±11.8 | **293.2^b^±9.6** | **225.2^b^±12.7** | 157.4 ^a^±10.1 |
|  | B2 | 282.2^a^±10.6 | **276.1^b^±9.6** | **298.3^c^±10.3** | 180.5 ^a^±11.2 |
| Wf |  |  |  |  |  |
| [g·plant^-1^] | NI | 6.89^a^±0.33 | 5.15^a^±0.10 | 1.26 ^a^±0.18 | 1.27 ^a^±0.06 |
|  | B1 | 6.66^a^±0.24 | **7.54^b^±0.39** | **4.17 ^b^±0.36** | **1.66 ^b^±0.17** |
|  | B2 | 6.27^a^±0.53 | 5.74 ^a^±0.39 | **4.37 ^b^±0.35** | 1.59 ^a^±0.13 |
|  |  |  |  |  |  |
| Wd  [g·plant^-1^] | NI | 0.71^b^±0.03 | 0.60^a^±0.03 | 0.19 ^a^±0.02 | 0.19 ^a^±0.01 |
|  | B1 | **0.84^c^±0.03** | **0.77^b^±0.03** | **0.42 ^b^±0.03** | 0.21 ^a^±0.02 |
|  | B2 | 0.57^a^±0.04 | 0.58^a^±0.04 | **0.44 ^b^±0.04** | 0.23 ^a^±0.02 |
|  |  |  |  |  |  |
| SLA  [cm^2^·g^-1^] | NI | 91.6^a^±4 | 100^a^±4 | 194 ^a^±69 | 158 ^a^±13 |
|  | B1 | 81.9^a^±6 | 91.1^a^±20 | 113 ^a^±10 | 139 ^a^±19 |
|  | B2 | 82.3^a^±15 | 118^a^±8 | 121 ^a^±7 | 144 ^a^±21 |
|  |  |  |  |  |  |
| LWR  [g·g^-1^] | NI | 0.51^a^±0.01 | 0.52 ^a^±0.01 | 0.51 ^a^±0.01 | 0.52^a^±0.01 |
|  | B1 | 0.46^a^±0.01 | 0.54 ^ab^±0.02 | 0.50 ^a^±0.01 | 0.46^a^±0.01 |
|  | B2 | **0.56^b^±0.07** | **0.57 ^b^±0.00** | **0.55^b^±0.01** | 0.51^a^±0.01 |
|  |  |  |  |  |  |
| RWR  [g·g^-1^] | NI | 0.093^a^±0.00 | 0.114 ^a^±0.00 | 0.099^a^±0.01 | 0.088 ^a^±0.01 |
|  | B1 | 0.124^a^±0.00 | 0.117 ^a^±0.01 | 0.095^a^±0.00 | **0.115^b^±0.01** |
|  | B2 | 0.138^a^±0.02 | 0.098 ^a^±0.01 | 0.106^a^±0.01 | **0.116^b^±0.01** |
|  |  |  |  |  |  |
| CCI | NI | 13.2^b^±0.33 | 15.7 ^a^±0.69 | 14.8^a^±1.8 | 7.4±1.8 |
|  | B1 | **16.0^c^±0.97** | 13.1 ^a^±0.39 | - | - |
|  | B2 | 10.3^a^±0.33 | 17.0 ^a^±0.45 | 15.5^a^±0.53 | - |
|  |  |  |  |  |  |
| TWC | NI | 88.8^b^±0.27 | 87.3 ^a^±0.39 | 85.8 ^a^±0.43 | 87.2 ^b^±0.26 |
|  | B1 | 86.9^a^±0.28 | **89.5 ^b^±0.32** | **88.9 ^b^±0.35** | 85.8 ^a^±0.38 |
|  | B2 | **89.7^c^±0.16** | **89.7 ^b^±0.24** | **88.4 ^b^±0.15** | 85.5 ^a^±0.37 |

NoL – number of leaves, RL – root length, W_f_ – fresh weight, W_d_ – dry weight, SLA – specific leaf area, LWR – leaf weight ratio, RWR – root weight ratio, TWC – tissue water content, CCI – chlorophyll content index, ns –not significant. Significant differences between NI, B1 and B2 variants in each NaCl solution are denoted by different letters (one-way ANOVA with Tuckey post-hoc comparisons). B1 and B2 significant positive effects are in bold, negative are underlined.

**Table 3.** Effect of endophytic bacteria inoculation (NI: non-inoculated, B1: inoculated with *P. stutzeri* ISE12, and B2: inoculated with *K. marisflavi* CSE9) on *Lactuca sativa* growth parameters in different NaCl concentrations. Means ± SE are presented.

|  | mMNaCl | | | | |
| --- | --- | --- | --- | --- | --- |
|  |  | 0 | 50 | 150 | 300 |
| NoL |  |  |  |  |  |
| [n] | NI | 9.1^a^ ±0.4 | 9.3 ^a^ ±0.4 | 8.9 ^a^ ±0.3 | - |
|  | B1 | 9.3 ^a^ ±0.3 | 9.3 ^a^ ±0.3 | 9.3 ^ab^±0.3 | 7.4 ^a^ ±0.2 |
|  | B2 | **11.0 ^b^ ±0.3** | **11.2 ^b^ ±0.4** | **10.4 ^b^ ±0.4** | 7.8 ^a^ ±0.5 |
| RL |  |  |  |  |  |
| [mm] | NI | 101.5 ^b^ ±5.0 | 86.2 ^ab^ ±9.3 | 62.1 ^ab^ ±8.0 | - |
|  | B1 | 65.1 ^a^ ±5.2 | 70.7 ^a^ ±5.7 | 44.9 ^a^ ±5.0 | 17.2 ^a^ ±2.2 |
|  | B2 | **129.5 ^c^ ±6.7** | 104.5 ^b^ ±7.6 | 85.0 ^b^ ±8.1 | **61.0 ^b^ ±8.2** |
| Wf |  |  |  |  |  |
| [g·plant^-1^] | NI | 3.03 ^a^ ±0.31 | 2.16 ^a^ ±0.18 | 0.84 ^a^ ±0.13 | - |
|  | B1 | 2.03 ^a^ ±0.28 | 1.69 ^a^ ±0.21 | 0.90 ^a^ ±0.20 | 0.17 ^a^ ±0.06 |
|  | B2 | **4.57 ^b^ ±0.34** | **4.73 ^b^ ±0.22** | **2.53 ^b^ ±0.39** | **0.85 ^b^ ±0.20** |
| Wd |  |  |  |  |  |
| [g·plant^-1^] | NI | 0.16 ^a^ ±0.01 | 0.12 ^a^ ±0.02 | 0.05 ^a^ ±0.01 | - |
|  | B1 | 0.10 ^a^ ±0.01 | 0.09 ^a^ ±0.01 | 0.06 ^a^ ±0.01 | 0.01 ^a^ ±0.00 |
|  | B2 | **0.33 ^b^ ±0.03** | **0.26 ^b^ ±0.02** | **0.12 ^b^ ±0.02** | 0.11 ^a^ ±0.07 |
| SLA |  |  |  |  |  |
| [cm^2^·g^-1^] | NI | 257 ^a^ ±28 | 376 ^b^ ±29 | 316 ^a^ ±23 | - |
|  | B1 | **409 ^b^ ±48** | 384 ^b^ ±41 | 187 ^a^ ±31 | 1698 ^a^ ±1360 |
|  | B2 | 207 ^a^ ±16 | 193 ^a^ ±29 | 262 ^a^ ±51 | 152 ^a^ ±47 |
| LWR |  |  |  |  |  |
| [g·g^-1^] | NI | 0.82 ^b^ ±0.02 | 0.84 ^c^ ±0.00 | 0.85 ^a^ ±0.01 | - |
|  | B1 | 0.80 ^b^ ±0.02 | 0.82 ^b^ ±0.01 | 0.85 ^a^ ±0.01 | 0.82 ^a^ ±0.11 |
|  | B2 | 0.72 ^a^ ±0.02 | 0.78 ^a^ ±0.01 | 0.80 ^a^ ±0.02 | 0.82 ^a^ ±0.05 |
| RWR |  |  |  |  |  |
| [g·g^-1^] | NI | 0.070 ^b^ ±0.01 | 0.078 ^a^ ±0.01 | 0.062 ^a^ ±0.01 | - |
|  | B1 | 0.041 ^a^ ±0.00 | 0.061 ^a^ ±0.01 | 0.071 ^a^ ±0.01 | 0.041 ^a^ ±0.02 |
|  | B2 | **0.126 ^c^ ±0.01** | **0.129 ^b^ ±0.01** | **0.133 ^b^ ±0.02** | 0.120 ^a^ ±0.05 |
| CCI |  |  |  |  |  |
|  | NI | 1.77 ^b^ ±0.04 | 2.04 ^b^ ±0.05 | 1.83 ^a^ ±0.06 | - |
|  | B1 | 1.23 ^a^ ±0.04 | 1.49 ^a^ ±0.06 | 1.79 ^a^ ±0.07 | 1.58 ^a^ ±0.14 |
|  | B2 | 1.90 ^b^ ±0.06 | 2.07 ^b^ ±0.06 | **2.55 ^b^ ±0.10** | **2.61 ^b^ ±0.08** |
| TWC |  |  |  |  |  |
| [%] | NI | 94.0 ^b^ ±0.46 | 94.1 ^b^ ±0.15 | 93.5 ^a^ ±0.18 | - |
|  | B1 | 95.0 ^b^ ±0.17 | 94.5 ^b^ ±0.14 | 92.8 ^a^ ±0.22 | 92.0 ^a^ ±1.29 |
|  | B2 | 92.6 ^a^ ±0.33 | 92.2 ^a^ ±0.33 | 93.5 ^a^ ±0.34 | 87.0 ^a^ ±6.03 |

NoL – number of leaves, RL – root length, W_f_ – fresh weight, W_d_ – dry weight, SLA – specific leaf area, LWR – leaf weight ratio, RWR – root weight ratio, TWC – tissue water content, CCI – chlorophyll content index, ns –not significant. Significant differences between NI, B1 and B2 variants in each NaCl solution are denoted by different letters (one-way ANOVA with Tuckey post-hoc comparison). B1 and B2 significant positive effects are in bold, negative are underlined.

**Table 4.** Effect of endophytic bacteria inoculation (NI: non-inoculated, B1: inoculated with *P. stutzeri* ISE12, and B2: inoculated with *K. marisflavi* CSE9) on *Helianthus annuus* growth parameters in different NaCl concentrations. Means ± SE are presented.

|  | mM NaCl | | | | |
| --- | --- | --- | --- | --- | --- |
|  |  | 0 | 50 | 150 | 300 |
| NoL |  |  |  |  |  |
| [n] | NI | 17.45 ^b^ ±0.52 | 16.60 ^ab^ ±0.41 | 16.30 ^a^ ±0.36 | 13.59 ^a^ ±0.42 |
|  | B1 | 15.58 ^a^ ±0.53 | 15.94 ^a^ ±0.37 | 15.20 ^a^ ±0.69 | 13.94 ^a^ ±0.47 |
|  | B2 | 18.15 ^b^ ±0.36 | 17.30 ^b^ ±0.26 | **18.60 ^b^ ±0.48** | **16.24 ^b^ ±0.55** |
| RL |  |  |  |  |  |
| [mm] | NI | 229 ^a^ ±14 | 367 ^ab^ ±43 | 274 ^ab^ ±15 | 151 ^b^ ±17 |
|  | B1 | 237 ^a^ ±33 | 265 ^a^ ±28 | 223 ^a^ ±15 | 96.4 ^a^ ±10 |
|  | B2 | **387 ^b^ ±41** | 443 ^b^ ±28 | 334 ^b^ ±26 | **198 ^c^ ±7.4** |
| Wf |  |  |  |  |  |
| [g·plant^-1^] | NI | 21.9 ^a^ ±2.2 | 22.0 ^a^ ±2.7 | 16.6 ^a^ ±0.91 | 9.11 ^a^ ±1.7 |
|  | B1 | 21.0 ^a^ ±2.0 | 23.2 ^a^ ±2.7 | 18.8 ^a^ ±1.7 | 7.86 ^a^ ±1.1 |
|  | B2 | **31.5 ^b^ ±1.2** | **25.6 ^b^ ±1.4** | **29.5 ^b^ ±3.4** | **13.9 ^b^ ±1.1** |
| Wd |  |  |  |  |  |
| [g·plant^-1^] | NI | 1.97 ^ab^ ±0.20 | 2.08 ^ab^ ±0.16 | 2.09 ^b^ ±0.18 | 0.85 ^a^ ±0.19 |
|  | B1 | 1.43 ^a^ ±0.20 | 1.62 ^a^ ±0.19 | 1.27 ^a^ ±0.13 | 0.45 ^a^ ±0.06 |
|  | B2 | 2.59 ^b^ ±0.14 | 2.25 ^b^ ±0.14 | **2.76 ^c^ ±0.23** | **1.39 ^b^ ±0.17** |
| SLA |  |  |  |  |  |
| [cm^2^·g^-1^] | NI | 72.3 ^a^ ±7.1 | 65.7 ^a^ ±6.5 | 41.7 ^a^ ±1.7 | 62.0 ^ab^ ±7.0 |
|  | B1 | 72.9 ^a^ ±6.7 | 65.3 ^a^ ±6.5 | **63.9 ^b^ ±6.1** | 74.8 ^b^ ±10.4 |
|  | B2 | 54.0 ^a^ ±5.3 | 50.2 ^a^ ±3.1 | 44.0 ^a^ ±3.6 | 36.8 ^a^ ±3.5 |
| LWR |  |  |  |  |  |
| [g·g^-1^] | NI | 0.22 ^a^ ±0.01 | 0.21 ^a^ ±0.01 | 0.23 ^a^ ±0.01 | 0.35 ^ab^ ±0.01 |
|  | B1 | **0.30 ^b^ ±0.01** | **0.29 ^b^ ±0.01** | **0.29 ^b^ ±0.01** | 0.37 ^b^ ±0.01 |
|  | B2 | 0.21 ^a^ ±0.01 | 0.21 ^a^ ±0.01 | 0.23 ^a^ ±0.01 | 0.32 ^a^ ±0.01 |
| RWR |  |  |  |  |  |
| [g·g^-1^] | NI | 0.087 ^b^ ±0.01 | 0.096 ^b^ ±0.01 | 0.102 ^b^ ±0.00 | 0.076 ^a^ ±0.01 |
|  | B1 | 0.059 ^a^ ±0.01 | 0.068 ^a^ ±0.00 | 0.078 ^a^ ±0.00 | 0.072 ^a^ ±0.01 |
|  | B2 | 0.093 ^b^ ±0.00 | 0.111 ^b^ ±0.01 | **0.117 ^c^ ±0.00** | **0.100 ^b^ ±0.01** |
| CCI |  |  |  |  |  |
|  | NI | 7.26 ^a^ ±0.27 | 7.24 ^a^ ±0.25 | 16.58 ^b^ ±0.64 | 20.67 ^b^ ±0.67 |
|  | B1 | **11.66 ^c^ ±0.34** | **11.00 ^c^ ±0.37** | 13.24 ^a^ ±0.35 | 13.70 ^a^ ±0.38 |
|  | B2 | **10.42 ^b^ ±0.27** | **9.44 ^b^ ±0.21** | 16.83 ^b^ ±0.45 | **24.74 ^c^ ±0.48** |
| TWC |  |  |  |  |  |
| [%] | NI | 90.4 ^a^ ±0.34 | 89.2 ^a^ ±0.40 | 87.7 ^a^ ±0.48 | 89.8 ^b^ ±0.86 |
|  | B1 | **92.6 ^b^ ±0.52** | **91.8 ^b^ ±0.43** | **91.0 ^b^ ±0.33** | **92.9 ^c^ ±0.12** |
|  | B2 | 90.7 ^a^ ±0.33 | 90.5 ^ab^ ±0.30 | 88.5 ^a^ ±0.47 | 87.2 ^a^ ±0.81 |

NoL – number of leaves, RL – root length, W_f_ – fresh biomass, W_d_ – dry biomass, SLA – specific leaf area, LWR – leaf weight ratio, RWR – root weight ratio, TWC – tissue water content, CCI – chlorophyll content index, ns –not significant. Significant differences between NI, B1 and B2 variants in each NaCl solution are denoted by different letters (one-way ANOVA with Tuckey post-hoc comparison). B1 and B2 significant positive effects are in bold, negative are underlined.
